# Preconception dietary patterns and time-to-conception in the high-income multi-country NiPPeR study

**DOI:** 10.1186/s12937-026-01283-0

**Published:** 2026-01-23

**Authors:** Jun S. Lai, Shan Xuan Lim, Sheila J. Barton, Elizabeth Huiwen Tham, Sarah El-Heis, Benjamin B. Albert, Caroline E. Childs, Cathryn A. Conlon, Marjorelee T. Colega, Vanessa Cox, Heidi Nield, See Ling Loy, Wayne S. Cutfield, Mary F.-F. Chong, Keith M. Godfrey, Shiao-Yng Chan

**Affiliations:** 1 Institute for Human Development and Potential, Agency for Science, Technology and Research, Singapore, 117609 Singapore; 2 Saw Swee Hock School of Public Health, National University of Singapore and National University Health System, Singapore, 117549 Singapore; 3 MRC Lifecourse Epidemiology Centre, University of Southampton, Southampton, SO17 1BJ UK; 4 NIHR Southampton Biomedical Research Centre, University of Southampton and University Hospital Southampton National Health Service Foundation Trust, Southampton, SO17 1BJ UK; 5 Department of Paediatrics, Yong Loo Lin School of Medicine, National University of Singapore and National University Health System, Singapore, 119228 Singapore; 6 Liggins Institute and A Better Start – National Science Challenge, The University of Auckland, Auckland, 1023 New Zealand; 7 Human Development and Health, Faculty of Medicine, University of Southampton, Southampton, SO16 6YD UK; 8 College of Health, Massey University, Palmerston North, 4442 New Zealand; 9 Department of Reproductive Medicine, KK Women’s and Children’s Hospital, Singapore, 229899 Singapore; 10 Obstetrics and Gynaecology Academic Clinical Program, Duke-NUS Medical School, Singapore, 169857 Singapore; 11 Department of Obstetrics & Gynaecology, Yong Loo Lin School of Medicine, National University of Singapore and National University Health System, Singapore, 119228 Singapore

**Keywords:** Preconception, Dietary patterns, Time-to-conception, Fertility, NiPPeR trial

## Abstract

**Background:**

Dietary patterns rich in plant-based foods, fish, and healthier fats are reportedly beneficial for fertility, but forming generalizable recommendations has been hindered by the lack of studies examining dietary patterns and time-to-conception (TTC) in cohorts with different ethnicities across geographical regions. To study the association of preconception dietary patterns with TTC in the multi-country NiPPeR trial.

**Methods:**

This study is a secondary analysis of data collected in the NiPPeR randomized controlled trial. Women planning to conceive, without known fertility impairment, were recruited from the community in the UK, Singapore, and New Zealand (NZ). Dietary intake was assessed at preconception prior randomization, and across-site (“pooled”) data-driven dietary patterns were derived (*n* = 1406). TTC, derived as the number of days between recruitment and the estimated date of achieving a clinical pregnancy, and the chance of achieving a clinical pregnancy within a year, expressed as hazard ratios (HR), were analyzed using Cox proportional hazards models adjusted for preconception body mass index, age and gravidity.

**Results:**

Two pooled dietary patterns were identified: “Vegetables, Fruits and Nuts” (VFN), and “Fried potatoes, Processed meat and Sweetened beverages” (FPS). Compared with the lowest quartile of VFN score, those in the highest quartile took a shorter time to conceive [Days till 20% conceived (95% CI): 73.0 (60.6, 91.5) vs 166.5 (120.0, 229.5)], and showed a higher chance of conception within a year [HR (95% CI): 2.15 (1.66, 2.78)]. This difference was most evident in Singapore, where the overall adherence to a VFN diet was substantially lower than in the UK and NZ [median (IQR) VFN score (expressed as standard deviation scores): Singapore -0.88 (-1.11, -0.57), UK 0.45 (0.07, 0.92), NZ 0.47 (-0.02, 0.90)]. There was no association between the FPS diet and TTC in the cohort.

**Conclusion:**

Consuming a diet rich in vegetables, fruits and nuts may shorten TTC and improve the chances of conception, particularly in populations with low intakes of such foods.

**Clinical trial registration:**

ClinicalTrials.gov, identifier: NCT02509988, Universal Trial Number U1111-1171–8056. Registered on 16 July 2015.

**Supplementary Information:**

The online version contains supplementary material available at 10.1186/s12937-026-01283-0.

## Introduction

Over the past five decades, there has been a steep decline in the global total fertility rate [1], most markedly in high-income countries. In the United Kingdom (UK), Singapore and New Zealand (NZ), fertility rates are below the two children per woman needed to maintain a stable population size [1]. This decline is partially contributed by subfertility, defined as the inability to conceive within 12 months of unprotected intercourse [2]. As such, identifying the modifiable factors associated with enhanced fertility is of great interest to increase chances of natural conception. Time-to-conception (TTC) is often used as a proxy for infertility in the absence of biomarkers, and couples are usually advised to seek fertility treatment after 12 months of regular, unprotected sexual intercourse [3]; reducing TTC can therefore be an important strategy for enhancing fertility rates.

A particular focus is the role of nutrition, with increasing evidence that consumption of more plant foods (fruits, vegetables, plant proteins), healthier fats (mono- or poly-unsaturated fats), and foods with a lower glycemic index enhance female fertility [4, 5]. However, there is limited recent literature examining dietary patterns and female fertility [6], with majority of studies examining single foods or nutrients [4, 5]. Examination of dietary patterns accounts for the combined and potentially synergistic effects of foods and nutrients on health and supports practical dietary recommendations as foods and nutrients are not consumed in isolation.

Diets high in fruits and vegetables, whole grains, nuts and legumes, and fish, and a reduction in the consumption of highly processed foods have been associated with lower odds of subfertility and ovulatory disorders [7, 8]. However, few studies have related dietary patterns to TTC or chance of conception in populations without known fertility impairment. In preconception studies, greater adherence to the Mediterranean Diet, Fertility Diet, Healthy Eating Index and healthful Plant-based Diet index – dietary patterns characterized by higher intakes of plant foods, healthier fats and/or fish, and less meat – have been associated with an increased chance of conception without ART in women without known fertility impairment [9, 10]. However, these studies used country-specific dietary data, thus producing findings which are population-specific, limiting application to other populations. Harmonization of dietary data across heterogeneous populations (e.g. multi-geographic and multi-ethnic) has become increasingly common as this method allows identification of common dietary patterns across countries and ethnicities [11], together with unique dietary patterns that may be beneficial for health. Unlike meta-analyses which synthesize study estimates derived independently across cohorts, harmonization of dietary data involve standardizing food groups definitions and the approach to derive dietary patterns, ensuring greater comparability across cohorts [12]. Findings generated from studies using harmonized dietary data could inform both globally-applicable and country-specific preconception dietary recommendations to improve fertility.

Using data from the NiPPeR (Nutritional Intervention Preconception and During Pregnancy to Maintain Healthy Glucose Metabolism and Offspring Health) study, we have previously harmonized and pooled baseline dietary data from women planning pregnancy across the three study sites, i.e. UK, Singapore and NZ [13]. We identified dietary patterns which were common to participants across the three sites, alongside patterns unique to each site [13]. In this study we examined the association of pooled and site-specific preconception dietary patterns with TTC.

## Materials and methods

### Study design and participants

The present study used data from the NiPPeR study, with previously published details [14, 15]. In brief, NiPPeR was a double-blind, randomized controlled trial (RCT) comparing the effects of a standard micronutrient supplement (control supplement: folic acid, β-carotene, iron, calcium, iodine) with an intervention supplement (study supplement) which further contained myo-inositol, additional micronutrients (vitamins B2, B6, B12, D, zinc) and probiotics (Lacticaseibacillus rhamnosus, Bifidobacterium animalis). The primary outcome of gestational glycemia demonstrated no difference between study groups [14, 15]. Women in the UK, Singapore and NZ, aged 18 to 38 years, who were planning to conceive were recruited from the community between 2015 and 2017. Upon enrolment, women were randomly assigned by an electronic database to either the control or study supplement in 1:1 ratio, stratified by site and ethnicity. Women consumed either supplement twice daily from preconception and throughout pregnancy. Women were not eligible to participate if they: 1) had diabetes mellitus (any type) or a severe allergy, 2) were pregnant or lactating at recruitment, 3) received contraception (oral, implanted or intrauterine), metformin, systemic steroids, anti-convulsant medication or treatment for HIV or Hepatitis B/C in the past month. The NiPPeR study protocol was registered on 16 July 2015 (https://www.clinicaltrials.gov/ct2/show/NCT02509988).

### Ethics approval and consent to participate

All procedures were approved by Research Ethics Committees at each study site [Southampton, UK: Health Research Authority, National Research Ethics Service Committee, South Central Research Ethics Committee (15/SC/0142); Singapore: National Healthcare Group Domain Specific Review Board (2015/00205); Auckland, NZ: Health and Disability Ethics Committee (15/NTA/21)], and conducted according to the Declaration of Helsinki for Medical Research. All participants provided written informed consent.

### Dietary assessment

Dietary intakes in the 1-month preceding recruitment were assessed at the respective study sites with validated, semi-quantitative food frequency questionnaires (FFQs) from the UK [16], SG [17] and NZ [18], and harmonized across all 3 study sites. Details on the harmonization of FFQs have been published [13]. The FFQs were administered in-person by trained research staff. For each FFQ item, participants were asked to indicate their frequency of consumption in an open-ended format (with options of Never, Frequency per month, Frequency per week, or Frequency per day) of standard portions of foods and beverages [13]. Total daily energy intakes were calculated for each participant using food composition database specific to each study site which reflects the dietary habits of the respective populations. To reduce measurement error resulting from self-reporting, women with implausible energy intakes (reported energy intake outside of a realistic range based on physiological status and physical activity level [19]), defined as energy intake of < 2092 kJ or > 29288 kJ (< 500 kcal or > 7000 kcal) using cut-offs determined a priori based on previous studies [20, 21], were excluded from subsequent analyses.

Dietary patterns were derived using factor analysis with varimax rotation. Both pooled (using 41 common food groups from all three study sites) and site-specific (using site-specific food groups) dietary pattern analyses were conducted, as previously published [13]. The number of patterns (i.e. factors) chosen to be retained was based on the break point of the scree plot, an eigenvalue > 1, and factor interpretability. Three dietary patterns were identified in pooled and site-specific analyses, with explained variances of 19–46%, 15–23% and 13–21%, respectively. Further details on the pooled and site-specific patterns including food items and their factor loadings have been published [13]. The first two dietary patterns identified from pooled and site-specific analyses were characterised by high intakes of similar foods: 1) the “Vegetables, Fruits and Nuts” (VFN) pattern (previously labelled as “Healthy”) was higher in vegetables and legumes (salad, root vegetables, peas, beans, legumes and pulses), fruits, and nuts; 2) the “Fried potatoes, Processed meat and Sweetened beverages” (FPS) pattern (previously labelled as “Less Healthy”) was higher in chips and fries, crisps and savoury snacks, processed meat, and sweetened beverages (Additional File 1). The pooled VFN and FPS pattern scores had high correlations with the respective pattern scores from each study site (*r* = 0.87–0.93) [13]; hence subsequent analyses for the first two dietary patterns utilised the pooled pattern scores. The third pattern differed across study sites and also from the pooled pattern (previously labelled as “Mixed”) – “Fish, Poultry, Noodles/Rice” identified for pooled, “Desserts, Pastries/Cakes, and Fried potatoes” identified for the UK, “Fish, Red meat, Mushroom, and Noodles” identified for Singapore, and “Fried snacks, Dried fruits, Fruit juices” identified for NZ [13]; hence subsequent analyses for the third pattern utilised site-specific pattern scores.

### Estimation of time-to-conception

When participants had a positive urinary pregnancy test, they were scheduled for an ultrasound scan to confirm a clinical pregnancy. Clinical pregnancy was defined as ultrasonographic evidence of a viable intra-uterine pregnancy with fetal cardiac activity detected after 6 weeks amenorrhea, including multiple pregnancies. Time-to-conception (TTC) of a clinical pregnancy without ART was calculated as the interval between the date of recruitment at preconception and the estimated date of conception (EDC). EDC was calculated as 38 weeks before the expected date of delivery (EDD), derived using an algorithm detailed in an earlier publication [22]. In brief, EDD was computed either from the first day of the last menstrual period (LMP) accounting for the individuals’ usual cycle length, or by the first ultrasound scan when there was > 7 days discrepancy from the LMP date, uncertain LMP date, an irregular cycle, or when hormonal contraception was stopped within the 3 months prior to pregnancy.

### Covariate assessment

At recruitment preconception, in-person interviews were conducted with enrolled participants to obtain the following information: sociodemographic characteristics (e.g., age, highest education attained, ethnicity), menstrual and obstetric histories, smoking, physical activity (the number of days they engaged in moderate and vigorous physical activity in the past 7 days), overall sleep quality (measured using the Pittsburgh Sleep Quality Index (PSQI) with a global PSQI score > 5 representing poor sleep quality [23]), and psychological stress (in general, how much stress or pressure have you experience in your daily living in the last 4 weeks?” taken from the 12-Item Short-Form Health Survey [24]). Body mass index (BMI) was derived using measured weight and height.

### Statistical analysis

Time-to-conception was estimated using Kaplan–Meier analyses, with statistical significance assessed by the log rank test. Censoring was applied when a woman was lost to follow-up, reported no longer trying to conceive, withdrew voluntarily or for a medical reason, subsequently initiated fertility/ART treatments including clomiphene/letrozole or metformin, miscarried before a clinical pregnancy could be established, had an ectopic pregnancy, or had not conceived after a year (Additional File 2). The period of 1 year was set a priori as after which time couples would usually be seeking infertility treatment. Additionally, this censors couples with previously unknown infertility issues that are likely less amenable to improvement with maternal diet/supplementation.

For each dietary pattern, participants were categorised into quartiles of dietary pattern score for the whole cohort, and in separate analyses, also categorised into quartiles of dietary pattern score for each study site. An indicative time at which 20% of women achieved conception within each quartile is presented [22]. Cox proportional hazards modelling for achievement of a clinical pregnancy within a year was used to estimate the hazard ratio (HR) with 95% confidence interval (CI) across score quartiles for the whole cohort and separately for each study site. All models were adjusted for study site, preconception BMI group [non-overweight/obese, overweight, and obese using ethnic-specific thresholds [15]], age and gravidity (never pregnant, have had a previous pregnancy) which are clinically important prognostic factors of fertility based on the literature [25, 26], and based on model parsimony (i.e. lower values in Akaike and Bayesian Information Criteria). Sensitivity analyses included further adjustment for energy intake, education, menstrual irregularity, supplement groups, smoking, physical activity, poor sleep quality and psychological stress. Site-specific analyses (including dietary pattern*site interaction) were also performed due to differences in pooled dietary pattern scores across study sites. Effect modification by the study supplement were also examined (dietary pattern*supplement), and stratified analyses by groups receiving study and control supplements were performed. The above analyses were repeated for the site-specific third dietary pattern.

All analyses were performed using STATA 17 software (StataCorp. 2021. Stata Statistical Software: Release 17. College Station, TX: StataCorp LP). Statistical significance was considered when the two-tailed probability was < 0.05.

## Results

**Fig. 1:**
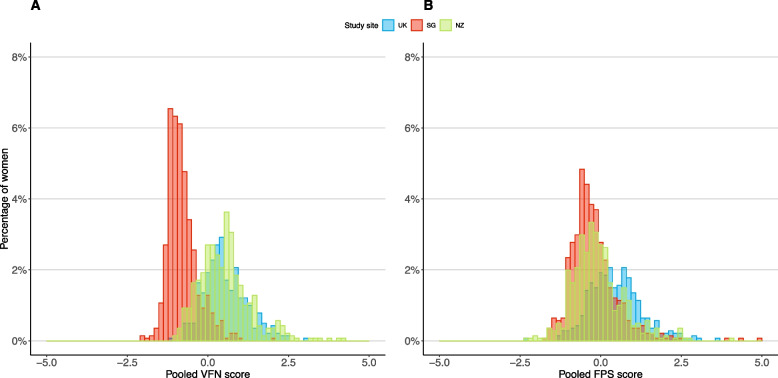
Preconception dietary pattern scores. Distributions of scores for (**A**) “Vegetables, Fruits and Nuts (VFN)” and (**B**) “Fried potatoes, Processed meat, and Sweetened beverages (FPS)” dietary patterns according to study sites. UK, United Kingdom.; NZ, New Zealand; SG, Singapore

A total of 1729 women were recruited and randomized; 1406 with both plausible energy intakes and data on TTC without ART were included in the current analysis (Additional File 2). The mean age of participants was 31 years. The majority were of White ethnicity in the UK (93.7%) and NZ (64.7%), and of Chinese ethnicity (64.4%) in Singapore (Table 1). Most women were highly educated (63.5% with degree), never conceived (52.5%), non-smokers (78.5%), and half of the women had overweight or obesity (50.6%). Of note, Singapore women had a substantially lower adherence to the VFN pattern (i.e. lower scores) than UK and NZ women; whilst UK women had a higher adherence to the FPS pattern (i.e. higher scores) than Singapore and NZ women (Fig. 1, Table 1).

**Table 1:** Characteristics of participants by study site in the analysis of preconception dietary patterns and time-to-conception in the NiPPeR cohort (*n* = 1406)^a^

	**All (*n* = 1,406)**	**UK (*n* = 363)**	**Singapore (*n* = 564)**	**NZ (*n* = 479)**
Age (years), mean (SD)	30.7 (3.6)	30.3 (4.0)	30.7 (3.5)	31.0 (3.4)
Race/ethnic origin, n (%)				
White	650 (46.2)	340 (93.7)	–-	310 (64.7)
Chinese	398 (28.3)	1 (0.3)	363 (64.4)	34 (7.1)
South Asian (Indian, Pakistani, Bangladeshi)	92 (6.5)	10 (2.8)	43 (7.6)	39 (8.1)
Malay	138 (9.8)	1 (0.3)	137 (24.3)	–-
Others (Polynesians, Blacks, other Asians)	128 (9.1)	11 (3.0)	21 (3.7)	96 (20.0)
Highest educational status, n (%)				
Below bachelor’s degree	513 (36.5)	170 (46.8)	215 (38.1)	128 (26.7)
Bachelor’s degree and above	893 (63.5)	193 (53.2)	349 (61.9)	351 (73.3)
Body mass index (BMI), n (%)				
Not overweight/obese	693 (49.4)	170 (46.8)	283 (50.4)	240 (50.1)
Overweight	374 (26.6)	103 (28.4)	145 (25.8)	126 (26.3)
Obese	337 (24.0)	90 (24.8)	134 (23.8)	113.0 (23.6)
	**All (*n* = 1,406)**	**UK (*n* = 363)**	**Singapore** **(*n* = 564)**	**NZ (*n* = 479)**
Smoking status, n (%)				
Never smoked	1101 (78.5)	245 (67.9)	486 (86.3)	370 (77.4)
Previously smoked	210 (15.0)	88 (24.4)	40 (7.1)	82 (17.2)
Actively smoking	91 (6.5)	28 (7.8)	37 (6.6)	26 (5.4)
Gravidity, n (%)				
No prior pregnancy	738 (52.5)	183 (50.4)	278 (49.3)	277 (57.8)
At least 1 prior pregnancy	668 (47.5)	180 (49.6)	286 (50.7)	202 (42.2)
Daily energy intake (kJ), mean (SD)	8636 (3029)	8058 (2201)	8263 (3151)	9510 (3238)
Dietary pattern scores, median (range)				
Vegetables, Fruit, Nuts	−0.03 (−2.06, 4.24)	0.45 (−1.09, 3.07)	−0.88 (−2.06, 1.97)	0.47 (−0.99, 4.24)
Fried potatoes, Processed meat, and Sweetened beverages	−0.13 (−2.34, 4.99)	0.38 (−2.34, 3.58)	−0.34 (−1.71, 4.99)	−0.20 (−2.25, 4.07)
Moderate or vigorous physical activity in the past week (days/wk), median (range)	3 (2, 5)	3 (1, 5)	2 (1, 5)	4 (2, 6)
	**All (*n* = 1,406)**	**UK (*n* = 363)**	**Singapore (*n* = 564)**	**NZ (*n* = 479)**
Psychological stress and pressure				
None	329 (23.4)	121 (33.3)	126 (22.3)	82 (17.1)
Slightly	701 (49.9)	155 (42.7)	295 (52.3)	251 (52.4)
Moderately to extremely	376 (26.7)	87 (24.0)	143 (25.4)	146 (30.5)

^a^*UK* United Kingdom, *NZ* New Zealand, *SG* Singapore

Missing values for BMI (*n* = 2), smoking status (*n* = 4), moderate or vigorous physical activity in the past week (*n* = 8)

### Association between pooled dietary patterns and time-to-conception

**Fig. 2:**
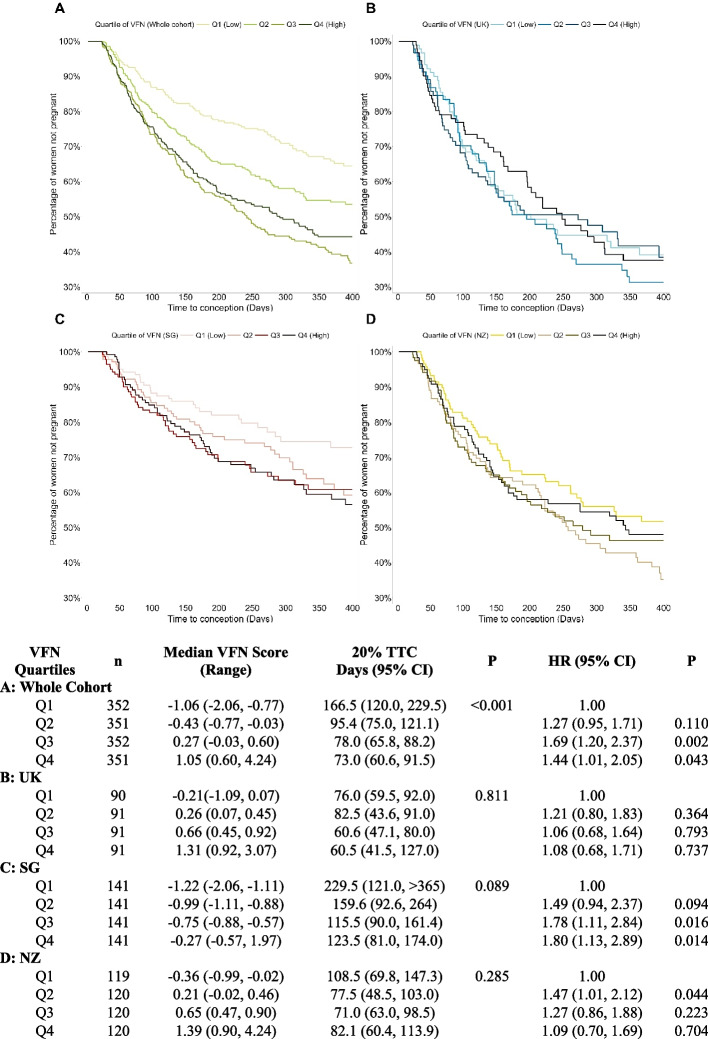
Kaplan Meier plots by quartiles of preconception dietary pattern. Time-to-conception (TTC) according to quartiles of “Vegetables, Fruits, and Nuts (VFN)” dietary pattern score of the whole cohort and each study site. Kaplan–Meier plot with over-time-censoring of withdrawn cases, including initiation of fertility treatment and very early pregnancy losses, for (**A**) whole cohort, (**B**) UK, (**C**) SG and (**D**) NZ. Hazard ratios (HR) by Cox proportional hazards modelling adjusted for energy intake, age, BMI, and gravidity. CI, Confidence Intervals; NZ, New Zealand; SG, Singapore UK; United Kingdom

The time taken for 20% of women to conceive was 73 days (95% CI: 60.6, 91.5) in the highest quartile (Q4) of the pooled VFN pattern score, compared with 166.5 days (120.0, 229.5) in the lowest quartile (Q1) (Fig. 2). The chance of conception within a year for women in Q3 and Q4 of the pooled VFN score were 1.69 times (95% CI: 1.20, 2.37) and 1.44 times (1.01, 2.05) that of women in Q1, respectively, after adjusting for site, age, BMI and gravidity (Fig. 2). Further adjustment for energy intake, education, menstrual irregularity, smoking, NiPPeR supplement, physical activity, poor sleep quality and psychological stress did not change the association (Additional File 3). There were no significant differences in VFN score [median (inter-quartile range): −0.078 (−0.772, 0.579) control vs −0.070 (−0.778, 0.615) study supplement; *P* = 0.424) as well as HRs according to groups receiving study or control supplements (Additional File 4).

The VFN-TTC relationship differed somewhat between study sites (*P*-interaction = 0.069), with the Singapore population showing a more accentuated pattern of association compared to the UK and NZ populations. In Singapore, the chances of conception within a year for women in Q3 and Q4 of the VFN scores were 1.78 times (95% CI: 1.11, 2.84) and 1.8 times (1.13, 2.89) that of women in Q1, respectively, after adjusting for age, BMI and gravidity (Fig. 2). In UK and NZ, there were no significant differences in HRs for higher quartiles (Q3 and Q4) of the VFN scores compared to Q1. The VFN-TTC relationship in the site-specific analyses did not differ by ethnicity (*P*-interactions > 0.1).

When considering the pooled FPS pattern, the time taken for 20% of women to conceive was similar, 84.5 days (71.4, 98.5) in Q4, compared with 91.5 days (73.0, 107.2) in Q1, and the HRs for achieving clinical pregnancy within a year showed no difference between Q4 and Q1 (Additional File 5). Particularly, in Singapore, the chance of conception within a year for women in Q3 of the pooled FPS score was lower, at 0.61 times (95% CI: 0.40, 0.92) that of women in Q1; however, in a post-hoc analysis, further adjusting for VFN adherence weakened the association (Q3 vs Q1: HR 0.66; 95% CI: 0.42, 1.04). There were no significant differences in FPS score [median (inter-quartile range): −0.12 (−0.54, 0.44) control vs −0.15 (−0.59, 0.45) study supplement; *P* = 0.477) according to groups receiving study or control supplements, and the interaction term (FPS*supplement) was not statistically significant (*P* = 0.499).

### Association between site-specific dietary patterns and time-to-conception

In Singapore, higher adherence to the “Fish, Red meat, Mushroom, Noodles” (FRMN) pattern was associated with a shorter TTC (Additional File 6); the time taken for 20% of women to conceive was 115.5 days (76.0, 167.5) in Q4, compared with 235 days (132.0, > 365) in Q1. The chances of conception for women in Q3 and Q4 of this pattern were 1.62 (95% CI: 1.02, 2.57) and 1.68 (1.02, 2.75) times that of women in Q1. In a post-hoc analysis, further adjusting for VFN adherence showed minimal change (Q4 vs Q1: HR 1.59; 95% CI: 1.00, 2.48). Associations also did not differ by ethnicity (*P*-interaction = 0.422).

There were no differences in TTC or HRs of conception across quartiles of the “UK – Desserts, Pastries/Cakes, Fried potatoes” and the “NZ – Fried snacks, Dried fruits, Fruit juices” dietary patterns.

## Discussion

We have previously identified two pooled dietary patterns – the VFN and FPS patterns, which are common across the three NiPPeR study sites (UK, Singapore and NZ). In the present study, we showed that a higher adherence to the pooled VFN dietary pattern was associated with a shortened TTC and almost a 1.5 times higher chance of conceiving within a year in women without known fertility impairment. There was a suggestive trend of lower chances of conceiving with greater adherence to the pooled FPS dietary pattern in Singapore. Additionally, adherence to the Singapore-specific FRMN dietary pattern was also associated with a higher chance of conceiving without ART.

Across the three study sites comprising multiple ethnicities, we found that increasing adherence to a dietary pattern characterized by higher intakes of vegetables and legumes, fruits, and nuts was associated with improved TTC and chances of conception. Our finding adds to existing literature showing that higher consumption of plant-based foods may improve fertility [4, 9], and further demonstrate that this association holds true across three different countries and multiple ethnicities. A diet high in vegetables and fruits is rich in antioxidants and has anti-inflammatory properties that can potentially counteract the oxidative stress and inflammation associated with reduced fertility [5, 9]. Although the mechanisms of action are unclear, oxidative stress and inflammation have been shown to affect oocyte quality and associate with ovulatory dysfunction and endometrial damage [27], hence affecting reproductive success. A higher intake of nuts may reflect higher consumption of mono-unsaturated fatty acids, which have also been reported to be beneficial for fertility, possibly through its binding to the peroxisome proliferator–activated receptor γ (PPAR-γ) and decreasing inflammation [4].

Interestingly, we observed that the improved chance of conceiving with a higher adherence to the VFN pattern was particularly evident among Singaporean women. In the Singaporean population, the range of VFN adherence was substantially lower than the adherence observed in the UK and NZ populations which may suggest lower intakes of such foods among Singaporean women or reflect differing dietary habits that influence comparability of the food groups characterizing the VFN pattern. We postulate that the impact of a VFN diet on conception may have reached saturation in UK and NZ. The median VFN score of the highest quartile (Q4) in Singapore is in a similar ballpark to the median VFN score of the lowest quartile (Q1) in UK and NZ. Thus, any improvement in adherence to the VFN pattern in Singapore could possibly still confer benefits on conception, whilst the benefits of further increasing adherence to the VFN pattern may be likely minimal for UK and NZ women who already have a relatively high VFN score.

A novel finding is a higher conception rate among Singaporean women with a higher adherence to the FRMN dietary pattern, a pattern which was present only in Singapore. This finding is supported by the long-assumed association between fish consumption and chances of live birth [28], although previous studies have been primarily in participants receiving infertility treatment. It has been postulated that omega-3 fatty acids have a beneficial effect on the growth and maturation of oocytes, can decrease the risk of anovulation, improve embryo development, and are associated with higher concentrations of progesterone which is key to sustaining pregnancy [29]. In addition to fish, this dietary pattern is also characterized by higher intakes of mushrooms, leafy vegetables (different from salads, root vegetables, peas/green beans in VFN pattern) and soy products [13], commonly consumed among Asians and constitute key foods characterizing dietary patterns favoring generally better health outcomes in Asian populations [30, 31]. Taken together, these findings suggest that dietary patterns characterized by healthier foods such as fruits and vegetables, nuts and legumes (including soy products), and fish can be beneficial for fertility, regardless of whether they are part of Asian or non-Asian diets. Future studies on the underlying biological mechanisms (e.g. inflammation and endocrine pathways) and integrating biomarker data (e.g., reproductive hormones, micronutrient levels) can help to better understand how the FRMN dietary pattern influences TTC.

Further analysis exploring differential effects between groups receiving the study or control supplements showed that the nutritional supplement did not enhance the beneficial influence of the VFN pattern on TTC or modify the associations of FPS pattern with TTC. This is in line with a previous publication showing no overall effects of the study supplement in improving TTC [22]. This finding highlights the merit of examining overall diets on health outcomes and the importance of improving dietary patterns as opposed to increasing intakes/levels of individual nutrients alone. Nonetheless, our previous publication reported that the study supplement shortened TTC particularly among women who were overweight, but we did not observe any difference in VFN-TTC associations between BMI categories (VFN*BMI *P*-interaction = 0.628). It is possible that the shorter-term nutritional supplementation could only correct for specific pertinent micronutrient deficiencies or inflammatory state associated with being overweight [32], thereby improving TTC; but a well-balanced diet over a longer term is required for overall improvement in TTC and chances of conception.

There was no overall association between adherence to the FPS dietary pattern with chances of conceiving in the cohort. In Singapore, however, there is suggestive evidence that greater adherence to the FPS pattern (Q3 vs Q1) lowered chances of conception; this association was weakened after adjusting for VFN scores. Given this, the association between greater FPS adherence on TTC is likely confounded by the low VFN adherence among Singaporean women. The existing literature on greater adherence to a dietary pattern characterized by red/processed meat, sweetened beverages, fast foods, and refined grains and fertility remains mixed with the Singaporean S-PRESTO study reported that a dietary pattern high in fast food and sweetened beverages being associated with reduced fecundability (chance of conceiving within a menstrual cycle) [10], but two other studies among Spanish and Iranian women found no associations between similar patterns (“Western-type dietary pattern”) with clinical pregnancy or difficulty conceiving [7, 33]. Future studies are required to confirm the association between dietary patterns characterised by similar foods and TTC.

### Strengths and limitations

Strengths of this study include identifying common dietary patterns across three countries and multiple ethnic groups that may influence TTC and chances of conception. Although data-driven approaches are generally used to derive population-specific patterns, we have successfully utilised this approach for cross-site/population analyses, deriving pooled VFN and FPS patterns that were comparable to site-specific patterns; findings generated from relating the pooled patterns to our outcome of interest are thus applicable across countries and ethnic groups. Future research may benefit from adopting data harmonisation approaches, which would enable more comparable cross-population analyses and support the development of nutrition strategies with wider applicability. A further strength is the inclusion of women from the general population, comprising a range of ethnicities, who were generally healthy without established fertility issues, as opposed to previous literature largely conducted among the minority infertile populations seeking ART, or in subgroups with high infertility risk (e.g. polycystic ovary syndrome), or in women with health issues recruited from hospital settings. Our findings are thus applicable to the majority of women who are trying to conceive, with the potential for a large impact on general fertility rates. Furthermore, infertile women may have reproductive issues that cannot be addressed by dietary interventions such as structural anomalies of the female reproductive tract and their inclusion in such studies would dilute any observed effects. Limitations include recruiting women who were actively trying to conceive which may mean that they were already adopting a healthier diet/lifestyle, as such, it is possible that amongst a general population displaying a wider distribution of dietary adherence, associations with TTC may be even stronger. We did not clinically assess participants for female/male infertility or reproductive pathologies known to affect fertility but did censor those who did not conceive after one year, beyond which couples are classified as subfertile. The time taken to conceive prior to study entry was not recorded and therefore could not be accounted for in the calculation of TTC in the present analysis. As with any studies using data-driven approaches to derive dietary patterns, findings are not immediately translatable to absolute amounts of specific foods to consume to improve TTC and chances of conception.

## Conclusions

In conclusion, a greater adherence to a dietary pattern higher in vegetables, fruits and nuts may improve TTC and chances of conception, particularly among the Singapore population where VFN adherence spanned a substantially lower range than other sites. Additionally, a greater adherence to a dietary pattern rich in fish, meat, mushroom and noodles amongst Singapore women may also improve fertility. Further investigation is required in multi-country settings with mixed ethnic profiles to inform generalizable dietary recommendations.

## Supplementary Information

Supplementary Material 1.

Supplementary Material 2.

Supplementary Material 3.

Supplementary Material 4.

Supplementary Material 5.

Supplementary Material 6.

## Data Availability

Individual participant data may be shared with an appropriately qualified individual working in an appropriate institution where an institutional signatory can confirm the recipient’s adherence to relevant information safeguards stipulated in a formal Data Transfer Agreement. Reasonable requests can be made through Professor Nicholas Harvey (nch@mrc.soton.ac.uk), as Director of the MRC Lifecourse Epidemiology Centre.

## Abbreviations

ART: Assisted Reproductive Technology
BMI: Body Mass Index
CI: Confidence Interval
EDC: Estimated Date of Conception
EDD: Estimated Date of Delivery
FFQ: Food Frequency Questionnaire
FPS: “Fried potatoes, Processed meat, and Sweetened beverages” pattern
LMP: Last Menstrual Period
NZ: New Zealand
NiPPeR: Nutritional Intervention Preconception and During Pregnancy to Maintain Healthy Glucose Metabolism and Offspring Health
TTC: Time-to-conception
UK: United Kingdom
VFN: “Vegetables, Fruits and Nuts” pattern

## References

[1] United Nations Department of Economic and Social Affairs Population Division: World population prospects 2022: summary of results. New York: United Nations; 2022.

[2] Zegers-Hochschild F, Adamson GD, Dyer S, Racowsky C, de Mouzon J, Sokol R, et al. The international glossary on infertility and fertility care, 2017. Fertil Steril. 2017;108:393–406. 28760517 10.1016/j.fertnstert.2017.06.005

[3] Hong X, Yin J, Wang W, Zhao F, Yu H, Wang B. The current situation and future directions for the study on time-to-pregnancy: a scoping review. Reprod Health. 2022;19:150. 35752834 10.1186/s12978-022-01450-6 PMC9233796

[4] Skoracka K, Ratajczak AE, Rychter AM, Dobrowolska A, Krela-Kaźmierczak I. Female fertility and the nutritional approach: the most essential aspects. Adv Nutr. 2021;12:2372–86. 34139003 10.1093/advances/nmab068 PMC8634384

[5] Alesi S, Villani A, Mantzioris E, Takele WW, Cowan S, Moran LJ, Mousa A. Anti-inflammatory diets in fertility: an evidence review. Nutrients. 2022;14. 10.3390/nu14193914 PMC9570802 36235567

[6] Winter HG, Rolnik DL, Mol BWJ, Torkel S, Alesi S, Mousa A, et al. Can dietary patterns impact fertility outcomes? A systematic review and meta-analysis. Nutrients. 2023;15:2589. 37299551 10.3390/nu15112589 PMC10255613

[7] Toledo E, Lopez-del Burgo C, Ruiz-Zambrana A, Donazar M, Navarro-Blasco I, Martínez-González MA, et al. Dietary patterns and difficulty conceiving: a nested case-control study. Fertil Steril. 2011;96:1149–53. 21943725 10.1016/j.fertnstert.2011.08.034

[8] Chavarro JE, Rich-Edwards JW, Rosner BA, Willett WC. Diet and lifestyle in the prevention of ovulatory disorder infertility. Obstet Gynecol. 2007;110:1050–8. 17978119 10.1097/01.AOG.0000287293.25465.e1

[9] Willis SK, Hatch EE, Laursen ASD, Wesselink AK, Mikkelsen EM, Tucker KL, et al. Dietary patterns and fecundability in 2 prospective preconception cohorts. Am J Clin Nutr. 2022;116:1441–51. 36192441 10.1093/ajcn/nqac213 PMC9630871

[10] Lim SX, Loy SL, Colega MT, Lai JS, Godfrey KM, Lee YS, et al. Prepregnancy adherence to plant-based diet indices and exploratory dietary patterns in relation to fecundability. Am J Clin Nutr. 2022;115:559–69. 34626169 10.1093/ajcn/nqab344 PMC7612357

[11] de Souza RJ, Zulyniak MA, Desai D, Shaikh MR, Campbell NC, Lefebvre DL, et al. Harmonization of food-frequency questionnaires and dietary pattern analysis in 4 ethnically diverse birth cohorts. J Nutr. 2016;146:2343–50. 27708121 10.3945/jn.116.236729

[12] Karageorgou D, Lara Castor L, Padula de Quadros V, Ferreira de Sousa R. Harmonising dietary datasets for global surveillance: methods and findings from the Global Dietary Database. 2024;27:e47. 10.1017/S1368980024000211 PMC10882534 38238892

[13] Lim SX, Cox V, Rodrigues N, Colega MT, Barton SJ, Childs CE, Conlon CA, Wall CR, Cutfield WS, Chan S-Y, et al. Evaluation of preconception dietary patterns in women enrolled in a multisite study. Curr Dev Nutr. 2022;6. 10.1093/cdn/nzac106 PMC9817353 36628060

[14] Godfrey KM, Cutfield W, Chan SY, Baker PN, Chong YS. Nutritional Intervention Preconception and During Pregnancy to Maintain Healthy Glucose Metabolism and Offspring Health (“NiPPeR”): study protocol for a randomised controlled trial. Trials. 2017;18:131. 28320484 10.1186/s13063-017-1875-x PMC5359891

[15] Godfrey KM, Barton SJ, El-Heis S, Kenealy T, Nield H, Baker PN, Chong YS, Cutfield W, Chan S-Y. Myo-inositol, probiotics, and micronutrient supplementation from preconception for glycemia in pregnancy: NiPPeR international multicenter double-blind randomized controlled trial. Diabetes Care. 2021:dc202515. 10.2337/dc20-2515 PMC8132330 33782086

[16] Crozier SR, Inskip HM, Godfrey KM, Robinson SM. Dietary patterns in pregnant women: a comparison of food-frequency questionnaires and 4 d prospective diaries. Br J Nutr. 2007;99:869–75. 18005481 10.1017/S0007114507831746 PMC3091014

[17] Lim SX, Colega MT, M Ayob MNi, Robinson SM, Godfrey KM, Bernard JY, et al. Identification and reproducibility of dietary patterns assessed with a FFQ among women planning pregnancy. Public Health Nutr. 2021;24:2437–46. 33745499 10.1017/S1368980021001178 PMC10195484

[18] Sam CH, Skidmore P, Skeaff S, Parackal S, Wall C, Bradbury KE. Relative validity and reproducibility of a short food frequency questionnaire to assess nutrient intakes of New Zealand adults. Nutrients. 2020;12:619. 32120797 10.3390/nu12030619 PMC7146506

[19] Banna JC, McCrory MA, Fialkowski MK, Boushey C. Examining plausibility of self-reported energy intake data: considerations for method selection. Front Nutr. 2017;4:45. 28993807 10.3389/fnut.2017.00045 PMC5622407

[20] Okesene-Gafa K, Li M, Taylor RS, Thompson JMD, Crowther CA, McKinlay CJD, et al. A randomised controlled demonstration trial of multifaceted nutritional intervention and or probiotics: the healthy mums and babies (HUMBA) trial. BMC Pregnancy Childbirth. 2016;16:373. 27884128 10.1186/s12884-016-1149-8 PMC5123375

[21] Rhee JJ, Sampson L, Cho E, Hughes MD, Hu FB, Willett WC. Comparison of methods to account for implausible reporting of energy intake in epidemiologic studies. Am J Epidemiol. 2015;181:225–33. 25656533 10.1093/aje/kwu308 PMC4325679

[22] Chan SY, Barton SJ, Loy SL, Chang HF, Titcombe P, Wong JT, Ebreo M, Ong J, Tan KM, Nield H, et al. Time-to-conception and clinical pregnancy rate with a myo-inositol, probiotics and micronutrient supplement: secondary outcomes of the NiPPeR randomized trial. Fertil Steril. 2023. 10.1016/j.fertnstert.2023.01.047 36754158

[23] Buysse DJ, Reynolds CF 3rd, Monk TH, Berman SR, Kupfer DJ. The Pittsburgh sleep quality index: a new instrument for psychiatric practice and research. Psychiatry Res. 1989;28:193–213. 2748771 10.1016/0165-1781(89)90047-4

[24] Gill SC, Butterworth P, Rodgers B, Mackinnon A. Validity of the mental health component scale of the 12-item short-form health survey (MCS-12) as measure of common mental disorders in the general population. Psychiatry Res. 2007;152:63–71. 17395272 10.1016/j.psychres.2006.11.005

[25] van Noord-Zaadstra BM, Looman CW, Alsbach H, Habbema JD, te Velde ER, Karbaat J. Delaying childbearing: effect of age on fecundity and outcome of pregnancy. BMJ. 1991;302:1361. 2059713 10.1136/bmj.302.6789.1361 PMC1670055

[26] Steiner AZ, Jukic AMZ. Impact of female age and nulligravidity on fecundity in an older reproductive age cohort. Fertil Steril. 2016;105:1584-1588.e1581. 26953733 10.1016/j.fertnstert.2016.02.028 PMC4893975

[27] Agarwal A, Aponte-Mellado A, Premkumar BJ, Shaman A, Gupta S. The effects of oxidative stress on female reproduction: a review. Reprod Biol Endocrinol. 2012;10:49. 22748101 10.1186/1477-7827-10-49 PMC3527168

[28] Gaskins AJ, Chavarro JE. Diet and fertility: a review. Am J Obstet Gynecol. 2018;218:379–89. 28844822 10.1016/j.ajog.2017.08.010 PMC5826784

[29] Trop-Steinberg S, Gal M, Azar Y, Kilav-Levin R, Heifetz EM. Effect of omega-3 supplements or diets on fertility in women: a meta-analysis. Heliyon. 2024;10:e29324. 38628754 10.1016/j.heliyon.2024.e29324 PMC11019195

[30] Lim GH, Neelakantan N, Lee YQ, Park SH, Kor ZH, van Dam RM, et al. Dietary patterns and cardiovascular diseases in Asia: a systematic review and meta-analysis. Adv Nutr. 2024;15:100249. 39009489 10.1016/j.advnut.2024.100249 PMC11294752

[31] Odegaard AO, Koh WP, Butler LM, Duval S, Gross MD, Yu MC, et al. Dietary patterns and incident type 2 diabetes in Chinese men and women: the Singapore Chinese Health Study. Diabetes Care. 2011;34:880–5. 21330641 10.2337/dc10-2350 PMC3064045

[32] Chapela SP, Martinuzzi ALN, Llobera ND, Ceriani F, Gonzalez V, Montalvan M, et al. Obesity and micronutrients deficit, when and how to suplement. Food Agric Immunol. 2024;35:2381725.

[33] Jahangirifar M, Taebi M, Nasr-Esfahani MH, Askari GH. Dietary patterns and the outcomes of assisted reproductive techniques in women with primary infertility: a prospective cohort study. Int J Fertil Steril. 2019;12:316–23. 30291693 10.22074/ijfs.2019.5373 PMC6186288

