# Supplementary material for: Preconception dietary patterns and time-to-conception in the high-income multi-country NiPPeR study
Source: Nutr J. 2026 Jan 23;25:23. doi: 10.1186/s12937-026-01283-0 (PMC12910744; doi:10.1186/s12937-026-01283-0)
Supplement: Supplementary file 1 — Supplementary Material 1. [file 12937_2026_1283_MOESM1_ESM.docx]

**Additional File 1**: Food groups characterising the “Vegetables, Fruits and Nuts (VFN)” and “Fried potatoes, Processed meat, and Sweetened beverages (FPS)” dietary patterns (factor loadings ≥0.25) for whole cohort (pooled) and each study site.

| **Vegetables, Fruits and Nuts (VFN)** | | | |
| --- | --- | --- | --- |
| **Pooled** | **UK** | **Singapore** | **New Zealand** |
| -Salad  -Root vegetables  -Peas, green beans, legumes, and pulses  -Other vegetables and gourds  -Tomatoes  -Bananas  -Potatoes and starchy vegetables  -Yoghurt  -Cheese  -Apples and pears  -Grapes, berries, stone fruits, and tropical fruits  -Nuts  -Breakfast cereals  -Citrus fruits and fruit juices  -Leafy vegetables  -Hot beverages  -Dried and canned fruits  -Noodles and pasta  -Rice  -Wholemeal/multigrain/brown bread  -Oily fish, whitefish, shellfish, and other seafood | -Salad  -Root vegetables  -Peas, green beans, legumes, and pulses  -Other vegetables and gourds  -Tomatoes  -Yoghurt  -Apples and pears  -Grapes, berries, stone fruits, and tropical fruits  -Nuts  -Leafy vegetables  -Dried and canned fruits  -White bread  -Eggs | -Salad  -Root vegetables  -Peas, green beans, legumes, and pulses  -Other vegetables and gourds  -Tomatoes  -Bananas  -Potatoes and starchy vegetables  -Yoghurt  -Cheese  -Grapes, berries, stone fruits, and tropical fruits  -Nuts  -Breakfast cereals  -Citrus fruits and fruit juices  -Leafy vegetables  -Dried and canned fruits  -Oily fish, whitefish, shellfish, and other seafood  -Onions | -Salad  -Root vegetables  -Peas, green beans, legumes, and pulses  -Other vegetables and gourds  -Tomatoes  -Potatoes and starchy vegetables  -Cheese  -Nuts  -Leafy vegetables  -Eggs  -Avocado |
| **Fried potatoes, Processed meat, and Sweetened beverages (FPS)** | | | |
| **Pooled** | **UK** | **Singapore** | **New Zealand** |
| -Chips and fries  -Crisps and savory snacks  -Ham, bacon, sausage, and other processed meat  -Sweetened beverages  -Pastries and cakes  -Chocolate  -White bread  -Pizza  -Other meats (pork, lamb, beef)  -Sweet biscuits and cookies  -Potatoes and starchy vegetables  -Other vegetables and gourds  -Tofu/ beancurd/ vegetarian foods  -Sweet and savory spreads  -Savory biscuits and crackers  -Sweet biscuits and cookies  -Oily fish, whitefish, shellfish, and other seafood  -Buns | -Crisps and savory snacks  -Ham, bacon, sausage, and other processed meat  -Sweetened beverages  -Chocolate  -Other meats (pork, lamb, beef)  -Sweet biscuits and cookies  -Poultry  -Salad  -Diet drinks | -Chips and fries  -Crisps and savory snacks  -Ham, bacon, sausage, and other processed meat  -Sweetened beverages  -Pastries and cakes  -Chocolate  -White bread  -Pizza  -Ice cream  -Cheese  -Noodles and pasta | -Chips and fries  -Crisps and savory snacks  -Ham, bacon, sausage, and other processed meat  -Sweetened beverages  -White bread  -Pizza  -Other meats (pork, lamb, beef)  -Sweet biscuits and cookies  -Potatoes and starchy vegetables  -Poultry  -Buns |
