# Supplementary material for: Preconception dietary patterns and time-to-conception in the high-income multi-country NiPPeR study
Source: Nutr J. 2026 Jan 23;25:23. doi: 10.1186/s12937-026-01283-0 (PMC12910744; doi:10.1186/s12937-026-01283-0)
Supplement: Supplementary file 2 — Supplementary Material 2. [file 12937_2026_1283_MOESM2_ESM.docx]

**Additional File 2:** Flowchart of participants included in the dietary pattern and time-to-conception analysis. NZ, New Zealand; UK, United Kingdom

Excluded:

40 new type 2 diabetes discovered during testing at recruitment

11 taking clomiphene or letrozole

112 voluntary withdrawal / loss to follow-up / other

129 without time-to-conception data

31 without dietary data / reported implausible energy intake

1729 recruited & completed first preconception visit

UK (n=460)

Singapore (n=661)

NZ (n=608)

1406 included in analysis

UK (n=363)

Singapore (n=564)

NZ (n=479)

607 clinical pregnancies at six weeks’ gestation

UK (n=202)

Singapore (n=175)

NZ (n=230)

Censored:

70 Pregnancy loss or non-viable before/at six weeks’ gestation

6 Ectopic pregnancies

683 Conceived

UK (n=223)

Singapore (n=207)

NZ (n=253)

Censored at different times through the one-year of study:

82 Initiated fertility treatment

326 Voluntary withdrawal / loss to follow-up / no longer trying to conceive / other

315 Did not conceive at 1 year
