# Supplementary material for: Preconception dietary patterns and time-to-conception in the high-income multi-country NiPPeR study
Source: Nutr J. 2026 Jan 23;25:23. doi: 10.1186/s12937-026-01283-0 (PMC12910744; doi:10.1186/s12937-026-01283-0)
Supplement: Supplementary file 3 — Supplementary Material 3. [file 12937_2026_1283_MOESM3_ESM.docx]

| **Additional File 3:** Cox proportional hazards modelling examining “Vegetables, Fruits and Nuts” (VFN) dietary pattern and chance of conception within a year adjusted for additional covariates^1^ | | | | | | | | | | | | |
| --- | --- | --- | --- | --- | --- | --- | --- | --- | --- | --- | --- | --- |
|  | **Whole cohort (n=1297)** | | | **UK (n=350)** | | | **Singapore (n=543)** | | | **NZ (n=462)** | | |
| **VFN quartiles** | n^2^ | HR (95% CI) | *P* | n | HR (95% CI) | *P* | N | HR (95% CI) | *P* | n | HR (95% CI) | *P* |
| Q1 | 334 | 1.00 |  | 85 | 1.00 |  | 134 | 1.00 |  | 102 | 1.00 |  |
| Q2 | 328 | 1.50 (1.15, 1.96) | 0.003 | 89 | 1.25 (0.81, 1.93) | 0.314 | 135 | 1.34 (0.84, 2.15) | 0.142 | 99 | 1.40 (0.94, 2.10) | 0.143 |
| Q3 | 326 | 2.33 (1.80, 3.01) | <0.001 | 90 | 1.08 (0.68, 1.71) | 0.739 | 134 | 1.59 (0.98, 2.58) | 0.090 | 100 | 1.25 (0.82, 1.90) | 0.177 |
| Q4 | 309 | 2.08 (1.58, 2.74) | <0.001 | 86 | 1.11 (0.68, 1.79) | 0.685 | 139 | 1.64 (1.01, 2.64) | 0.046 | 103 | 1.06 (0.67, 1.71) | 0.621 |
| CI, Confidence Interval; HR, Hazard Ratio; NZ, New Zealand; UK, United Kingdom  ^1^ Adjusted for site (except site-specific analysis), age, BMI, gravidity, energy intake, education, menstrual irregularity, NiPPeR supplement, smoking, physical activity, psychological stress and poor sleep quality  ^2^ Missing values: n=21 menstrual irregularity, n=4 smoking, n=8 physical activity, n=76 sleep quality | | | | | | | | | | | | |
