# Supplementary material for: Preconception dietary patterns and time-to-conception in the high-income multi-country NiPPeR study
Source: Nutr J. 2026 Jan 23;25:23. doi: 10.1186/s12937-026-01283-0 (PMC12910744; doi:10.1186/s12937-026-01283-0)
Supplement: Supplementary file 4 — Supplementary Material 4. [file 12937_2026_1283_MOESM4_ESM.docx]

**Additional File 4**: Cox proportional hazards modelling^1^ examining “Vegetables, Fruits and Nuts” (VFN) dietary pattern and chance of conception within a year for the whole cohort stratified by supplement groups^2^.

|  | **Control supplement (n=684)** | | | **Study supplement (n=722)** | | |  |
| --- | --- | --- | --- | --- | --- | --- | --- |
| **VFN quartiles** | n | HR (95% CI) | P | n | HR (95% CI) | P |  |
| Q1 | 170 | 1.00 |  | 182 | 1.00 |  |  |
| Q2 | 182 | 1.17 (0.78, 1.77) | 0.441 | 169 | 1.37 (0.90, 2.09) | 0.147 |  |
| Q3 | 165 | 1.35 (0.83, 2.19) | 0.224 | 187 | 2.16 (1.35, 3.46) | 0.001 |  |
| Q4 | 167 | 1.28 (0.78, 2.11) | 0.329 | 184 | 1.71 (1.03, 2.82) | 0.036 |  |
|  |  |  |  |  | *P*-interaction | 0.545 |  |
| NiPPeR, Nutritional Intervention Preconception and During Pregnancy to Maintain Healthy Glucose Metabolism and Offspring Health trial; CI, Confidence Interval; HR, Hazard Ratio  ^1^ Adjusted for energy, site, age, BMI, gravidity  ^2^ Control supplement is a standard supplement containing folic acid, β-carotene, iron, calcium, iodine; the NiPPeR-trial study supplement contains the standard supplement ingredients plus vitamins B2, B6, B12, D, zinc, myo-inositol and probiotics (*Lacticaseibacillus rhamnosus*, *Bifidobacterium animalis*). | | | | | | | |
