# Supplementary material for: Preconception dietary patterns and time-to-conception in the high-income multi-country NiPPeR study
Source: Nutr J. 2026 Jan 23;25:23. doi: 10.1186/s12937-026-01283-0 (PMC12910744; doi:10.1186/s12937-026-01283-0)
Supplement: Supplementary file 5 — Supplementary Material 5. [file 12937_2026_1283_MOESM5_ESM.docx]

**Additional File 5**: Time to conception (TTC) and Hazard ratios (HR) by Cox proportional hazards modelling for conceiving within a year according to quartiles of “Fried potatoes, Processed meat, and Sweetened beverages (FPS)” dietary pattern scores of the whole cohort and each study site.

| **FPS quartiles** | **n** | **Median (Range)** | **20% TTC**  **Days (95% CI)** | **P trend** | **HR (95% CI)^1^** | **P** |
| --- | --- | --- | --- | --- | --- | --- |
| **Whole cohort (n=1406)** | | | | | | |
| Q1 | 352 | -1.06 (-2.06, -0.77) | 91.5 (73.0, 107.2) | 0.039 | 1.00 |  |
| Q2 | 351 | -0.43 (-0.77, -0.03) | 132.6 (99.5, 159.6) |  | 0.87 (0.68, 1.10) | 0.234 |
| Q3 | 352 | 0.27 (-0.03, 0.60) | 80.5 (67.5, 100.4) |  | 1.17 (0.93, 1.47) | 0.175 |
| Q4 | 351 | 1.05 (0.60, 4.24) | 84.5 (71.4, 98.5) |  | 1.22 (0.94, 1.57) | 0.129 |
| **UK (n=363)** | | | | | | |
| Q1 | 90 | -0.21(-1.09, 0.07) | 83.0 (43.6, 128.0) | 0.494 | 1.00 |  |
| Q2 | 91 | 0.26 (0.07, 0.45) | 64.5 (49.0, 97.6) |  | 1.04 (0.70, 1.56) | 0.833 |
| Q3 | 91 | 0.66 (0.45, 0.92) | 60.0 (38.0, 84.5) |  | 1.18 (0.78, 1.77) | 0.431 |
| Q4 | 91 | 1.31 (0.92, 3.07) | 78.2 (47.0, 95.0) |  | 0.91 (0.57, 1.47) | 0.711 |
| **SG (n=564)** | | | | | | |
| Q1 | 141 | -1.22 (-2.06, -1.11) | 102.5 (55.5, 156.0) | 0.089 | 1.00 |  |
| Q2 | 141 | -0.99 (-1.11, -0.88) | 151.0 (92.0, 194.0) |  | 0.75 (0.50, 1.11) | 0.151 |
| Q3 | 141 | -0.75 (-0.88, -0.57) | 189.0 (121.1, 268.0) |  | 0.61 (0.40, 0.92) | 0.020 |
| Q4 | 141 | -0.27 (-0.57, 1.97) | 166.5 (90.5, 279.0) |  | 0.68 (0.41, 1.12) | 0.127 |
| **NZ (n=479)** | | | | | | |
| Q1 | 119 | -0.36 (-0.99, -0.02) | 81.2 (64.3, 102.5) | 0.069 | 1.00 |  |
| Q2 | 120 | 0.21 (-0.02, 0.46) | 82.0 (65.8, 132.6) |  | 1.01 (0.69, 1.48) | 0.944 |
| Q3 | 120 | 0.65 (0.47, 0.90) | 68.3 (48.5, 95.5) |  | 1.42 (0.99, 2.03) | 0.056 |
| Q4 | 120 | 1.39 (0.90, 4.24) | 103.0 (70.1, 141.5) |  | 0.90 (0.58, 1.39) | 0.627 |

CI, Confidence Intervals; NZ, New Zealand; SG, Singapore UK; United Kingdom

^1^Adjusted for energy, site (except site-specific analysis), age, BMI, and gravidity.
