# Supplementary material for: Preconception dietary patterns and time-to-conception in the high-income multi-country NiPPeR study
Source: Nutr J. 2026 Jan 23;25:23. doi: 10.1186/s12937-026-01283-0 (PMC12910744; doi:10.1186/s12937-026-01283-0)
Supplement: Supplementary file 6 — Supplementary Material 6. [file 12937_2026_1283_MOESM6_ESM.docx]

**Additional File 6**: Time to conception (TTC) and Hazard ratios (HR) by Cox proportional hazards modelling for conceiving within a year according to quartiles of site-specific dietary pattern scores.

|  | **n** | **20% TTC**  **Days (95% CI)** | **P trend** | **HR (95% CI)^1^** | **P** |
| --- | --- | --- | --- | --- | --- |
| **UK (n=363) – Desserts, Pastries/Cakes, Fried potatoes** | | | | | |
| Q1 | 90 | 85.9 (42.8, 117.0) | 0.481 | 1.00 |  |
| Q2 | 91 | 76.0 (47.5, 86.5) |  | 1.26 (0.86, 1.85) | 0.233 |
| Q3 | 91 | 89.0 (57.7, 104.0) |  | 0.92 (0.61, 1.38) | 0.678 |
| Q4 | 91 | 53.0 (36.5, 74.0) |  | 1.10 (0.72, 1.69) | 0.638 |
| **SG (n=564) – Fish, Red meat, Mushroom, Noodles** | | | | | |
| Q1 | 141 | 235.0 (132.0, >365) | 0.048 | 1.00 |  |
| Q2 | 141 | 151.0 (85.5, 229.5) |  | 1.36 (0.85, 2.17) | 0.203 |
| Q3 | 141 | 127.4 (63.0, 186.5) |  | 1.62 (1.02, 2.57) | 0.039 |
| Q4 | 141 | 115.5 (76.0, 167.5) |  | 1.68 (1.02, 2.75) | 0.040 |
| **NZ (n=479) – Fried snacks, Dried fruits, Fruit juices** | | | | | |
| Q1 | 119 | 82.3 (70.0, 104.0) | 0.897 | 1.00 |  |
| Q2 | 120 | 102.4 (57.5, 130.5) |  | 0.90 (0.62, 1.32) | 0.588 |
| Q3 | 120 | 77.0 (63.0, 115.9) |  | 1.02 (0.69, 1.49) | 0.934 |
| Q4 | 120 | 74.0 (58.5, 100.0) |  | 1.06 (0.71, 1.58) | 0.784 |
| CI, Confidence Intervals; NZ, New Zealand; SG, Singapore UK; United Kingdom | | | | | |
| ^1^ Adjusted for energy, age, BMI, and gravidity | | | | | |
